# Longitudinal Study of Group B Streptococcus Carriage in Pregnancy

**DOI:** 10.1155/S1064744997000409

**Published:** 1997

**Authors:** Jean R. Goodman, Richard L. Berg, Robert K. Gribble, Paul R. Meier, Susan C. Fee, Paul D. Mitchell

**Affiliations:** 1 Departments of Obstetrics and Gynecology and Microbiology Marshfield Clinic 1000 N. Oak Avenue Marshfield WI 54449 USA; 2 Department of Epidemiology/Biostatistics Marshfield Medical Research Foundation Marshfield WI USA


Infectious Diseases in Obstetrics and Gynecology 5:237–243 (1997)
© 1997 Wiley-Liss, Inc.

### Longitudinal Study of Group B Streptococcus Carriage in Pregnancy

Jean R. Goodman,* Richard L. Berg, Robert K. Gribble, Paul R. Meier, Susan C. Fee, and Paul D. Mitchell

*Departments of Obstetrics and Gynecology and Microbiology, Marshfield Clinic, Marshfield, WI*
*Department of Epidemiology/Biostatistics, Marshfield Medical Research Foundation, Marshfield, WI*

### Abstract

***Objective:*** This prospective study was designed to 1) determine the prevalence of group B streptococcus (GBS) in our obstetric population and 2) evaluate the predictive value of lower vaginal/perianal GBS cultures obtained in each trimester of pregnancy relative to GBS culture status at delivery.

***Methods:*** Lower vaginal/perianal GBS cultures were obtained in the first trimester, at 26–28 weeks, at 37 weeks, and on admission for delivery. The investigators were blinded to the results of all cultures except those obtained at 37 weeks. The sensitivity, specificity, and positive (PPV) and negative predictive values (NPV) of each group of cultures with respect to culture status at delivery were determined, and the pattern of GBS carriage in our patients was delineated.

***Results:*** Nine hundred seventy-three patients participated in this longitudinal study. The prevalence of GBS carriage was 14.0% in the first trimester, 13.9% at 26–28 weeks, 12.4% at 37 weeks, and 12.1% at delivery. GBS carriage was continuous (all 4 cultures positive) in 3.8% and identified on a single culture only in 7.8%. Sensitivity (S1), specificity (S2), PPV, and NPV for each set of antepartum cultures with respect to culture status at delivery were as tabled:

| | Delivery GBS status (%) | | | |
|---|---|---|---|---|
| | S1 | S2 | PPV | NPV |
| First trimester | 49.5 | 91.8 | 45.5 | 92.9 |
| 26–28 weeks | 68.4 | 93.9 | 60.4 | 95.6 |
| 37 weeks | 63.3 | 94.5 | 61.3 | 95.0 |

***Conclusions:*** The pattern of GBS carriage in pregnancy is highly variable. Regardless of when antenatal GBS cultures are done, they serve as poor predictors of maternal GBS carriage at delivery. Infect. Dis. Obstet. Gynecol. 5:237–243, 1997. © 1997 Wiley-Liss, Inc.

Key words infection; cultures; antepartum; screening

The group B beta-hemolytic streptococcus (GBS) is recognized as a significant perinatal pathogen, being responsible for 1.8 cases of neonatal sepsis per 1,000 live births each year in the United States.[1,2] The vast majority of these neonatal infections are the result of peripartum vertical transmission from a GBS colonized mother.[3] Gastrointestinal GBS colonization has been recognized as

Presented in part at the Society of Perinatal Obstetricians 16th Annual Meeting, Kona, Hawaii, February 1996.

Contract grant sponsors: Perinatal Foundation, Madison, WI, and Marshfield Medical Research Foundation, Marshfield, WI.

*Correspondence to: Dr. Jean Ricci Goodman, Department of Obstetrics and Gynecology, Marshfield Clinic, 1000 N. Oak Avenue, Marshfield, WI 54449. E-mail: goodmaj@dgabby.mfldclin.edu

Received 10 January 1997

Accepted 25 April 1997

Clinical Study the major source of genital tract seeding in pregnancy, and lower vaginal colonization with GBS at the time of delivery represents the major determining factor of mother-to-infant transmission.[4]

The reported incidence of lower vaginal/perianal GBS colonization in pregnancy varies from 15 to 40%.[5–7] When maternal vaginal GBS colonization is present at delivery, approximately 70–75% of delivered infants will be colonized.[8] Of term infants colonized with GBS, 1–2% will develop a GBS-related infection, whereas preterm infants are at a significantly higher risk of infection with an 8% attack rate.[7,8] Reported neonatal mortality rates range from 5 to 20%, with the potential for lethal infection highest in preterm infants.[1,2] GBS is also a major cause of puerperal sepsis, being the most common isolate in chorioamnionitis and accounting for 20% of cases of endometritis.[9]

The prevention of GBS-related disease in pregnancy is dependent on the interruption of peripartal vertical transmission. Intrapartum therapy of GBS carriers with systemic antibiotics has been shown to markedly diminish both neonatal and maternal puerperal infection rates.[10,11] However, for a selective intrapartum approach to be effective, a good predictor of colonization at the time of delivery is needed. A number of rapid assays for the detection of GBS in labor have been developed but all lack sufficient sensitivity (70–80%) and require heavy GBS colonization to achieve even that sensitivity level.[11–13] Longitudinal studies on the predictive value of prenatal GBS cultures have shown varied results and differ in the site cultured.[3,5,6,8,14,15] Whether antenatal cultures would be useful to reliably determine who should receive intrapartum therapy has thus represented a controversy in the literature to date. In addition, if screening GBS cultures were to be routinely obtained, at what gestational age they would best be obtained is unclear.

The primary objective of this study was to evaluate the predictive value of vaginal/perianal GBS cultures obtained in each trimester relative to culture status at delivery. A second objective was to define the prevalence of GBS carriage in our population.

### SUBJECTS AND METHODS

Beginning June 15, 1993, all women seen for their first prenatal visit in the first trimester of pregnancy at the Marshfield Clinic, a tertiary care center located in central Wisconsin, were considered eligible for participation in this study. Recruitment occurred during the initial visit, and patients who agreed to participate had vaginal/perianal cultures done at their first visit, at 26–28 weeks, at 37 weeks, and on admission in labor. The investigators were blinded to the culture results, except for the result of the 37 week culture.

Culture specimens were obtained from the lower vaginal and perianal areas with a single Dacron swab, then placed in Amies transport medium and immediately transferred to the laboratory. The specimens were then plated directly on colistin nalidixic acid (CNA) agar with blood within 1 h of receipt in the laboratory. Because plating was done directly to this selective solid media, no subcultures were required. All isolates of streptococci were serotyped with antisera from Becton Dickinson (Cockeysville, MD). Bacterial growth on the agar plate was quantified based on the quadrant of the plate involved. Growth in the initial half of the plate was semiquantitated as light, into the third quadrant as moderate, and into the fourth quadrant as heavy.

Without prior knowledge of the prevalence of GBS carriage in our obstetric population, an estimate on required sample size to fulfill the study objectives was based on a presumed prevalence of 15%. A comparison of the sensitivity of two culture times with respect to culture status at delivery was estimated to require 946 patients to achieve a statistical power of 0.90 (assuming true sensitivities of 0.8 and 0.9, in a two-sided test with a significance level of 0.05). Since more than 1,100 new obstetric patients are seen each year at Marshfield Clinic, it was anticipated that enrollment would be completed in 1 year. The study proposal was approved by the Marshfield Clinic Institutional Review Board in May 1993.

After delivery of all patients, the culture data were analyzed to determine the prevalence of GBS carriage in each trimester and at delivery. The sensitivity, specificity, and positive (PPV) and negative predictive values (NPV) of cultures in each trimester with respect to culture status at delivery were determined. The prevalence of GBS carriage and the predictive values of cultures were summarized as percents, with 95% confidence limits. The primary statistical analyses comparing culture results at different time points were based upon ex-

TABLE 1. GBS study culture times and prevalence[a]

| | N | Time of culture[b] | Prevalence[c] |
|---|---|---|---|
| First trimester | 973 | 9.9 ± 2.6 | 14.0 (11.8, 16.3) |
| 26–28 weeks | 876 | 27.6 ± 1.1 | 13.9 (11.7, 16.4) |
| 37 weeks | 792 | 37.3 ± 0.5 | 12.4 (10.2, 14.9) |
| Delivery | 832 | 39.3 ± 1.9 | 12.1 (10.0, 14.6) |

[a]Based on all available cultures.
[b]Mean ± SD (weeks gestation).
[c]Percent (95% confidence limits).

TABLE 2. GBS study culture prevalence in patients with all 4 cultures

| | N | Prevalence[a] |
|---|---|---|
| First trimester | 735 | 13.2 (10.8, 15.9) |
| 26–28 weeks | 735 | 14.0 (11.6, 16.7) |
| 37 weeks | 735 | 12.5 (10.2, 15.1) |
| Delivery | 735 | 12.1 (9.8, 14.7) |

[a]Percent (95% confidence limits).

act results for McNemar's test of correlated proportions. Chi-square procedures were used to test for homogeneity of prevalence over age groups and other demographic characteristics. Multiple logistic regression analysis was used to jointly evaluate the association of GBS prevalence with the set of patient characteristics and to evaluate the use of ordinal grading for positive cultures. Results in this report are deemed statistically significant at the 5% level of significance ($P < 0.05$). Analyses were conducted using StatXact and SAS statistical software.

### RESULTS

Between June 15, 1993, and December 31, 1994, 1,302 patients presented for prenatal care during the first trimester at our center. Of these, 1,285 were offered the opportunity to participate in the study and 973 (76%) enrolled. The 27 additional patients were recruited in an attempt to compensate for 89 patients lost prior to the second culture. Continued recruitment to compensate for all 89 was not possible due to financial constraints. Enrollment ended December 31, 1994, and all enrolled patients were delivered by August 15, 1995.

As mentioned, 89 patients were lost from the study before the second (26–28 week) culture was obtained due to miscarriage (43), pregnancy termination (11), move/change of care (30), delivery (3), or patient decision to withdraw from the study (2). At the time of the 26–28 week culture, 884 patients remained, and of these 876 had their cultures done. Seven hundred ninety-seven patients remained undelivered by the 37 week appointment with 792 cultures obtained. Delivery cultures were obtained for 832 patients who delivered after 20 weeks. Overall, 735 subjects had a complete set of all 4 cultures to evaluate.

The mean gestational age of subject enrollment was 9.9 ± 2.6 weeks. The mean times at which study cultures were obtained and the corresponding GBS prevalence at these times based upon available cultures are summarized in Table 1. In Table 2, results are presented for those patients in whom all 4 study cultures were done. Although the observed GBS prevalence was slightly higher at the early culture times in comparison to the delivery culture, this difference was not statistically significant.

Table 3 presents the sensitivity, specificity, and predictive values for each culture time with respect to culture status at delivery. The observed sensitivities and PPVs were below 50% for the first trimester culture, and were only 60–70% at 26–28 and 37 weeks. For those patients found positive at delivery, comparisons of early culture results show a significant improvement in sensitivity from the first trimester to 26–28 and 37 weeks ($P = 0.004$ and $P = 0.017$, respectively), but no difference between the 26–28 and 37 week cultures ($P = 0.47$). Regardless of whether the antenatal culture was done at 26–28 or 37 weeks, PPV and sensitivity with respect to culture status at delivery did not significantly improve.

Prevalence data for GBS at the first trimester visit are presented in Table 4 stratified by various demographic characteristics. Almost all patients were white, 86% were currently married, and the mean age was 27.7 ± 5.3 years. A significant nonlinear association between prevalence and age was observed ($P = 0.008$), with the highest prevalence observed among women in their late 20s. Significantly lower prevalence was observed in women who had experienced a prior miscarriage (9.8% vs. 15.8%, $P = 0.012$) and among those women reporting tobacco use (8.49% vs. 15.3%, $P = 0.013$). Multiple logistic regression served to verify that age, prior miscarriage, and tobacco use were jointly associated with GBS prevalence.

Of the 735 patients with complete (all 4) culture data, cultures were consistently negative in 564 patients (76.7%). Of 171 patients (23.3%) who were

TABLE 3. GBS carriage rates with respect to culture status at delivery[a]

| | Sensitivity | Specificity | PPV | NPV |
|---|---|---|---|---|
| First trimester | 49.5 (39.4, 59.6) | 91.8 (89.6, 93.7) | 45.5 (35.9, 55.2) | 92.9 (90.8, 94.7) |
| 26–28 weeks | 68.4 (58.2, 77.4) | 93.9 (91.9, 95.5) | 60.4 (50.6, 69.5) | 95.6 (93.8, 97.0) |
| 37 weeks | 63.3 (52.5, 73.2) | 94.5 (92.5, 96.1) | 61.3 (50.6, 71.2) | 95.0 (93.0, 96.5) |

[a]Percent (95% confidence limits).

TABLE 4. Demographics of study population based on initial culture result[a]

| | GBS+ | GBS− | P* |
|---|---|---|---|
| Age (years) | | | 0.008 |
| ≤21 | 19 (13.1%) | 126 | |
| 22–25 | 18 (8.1%) | 201 | |
| 26–29 | 52 (19.1%) | 220 | |
| 30–33 | 35 (15.6%) | 190 | |
| ≥34 | 12 (10.7%) | 100 | |
| Married | | | |
| Currently | 117 (14.0%) | 716 | 0.897 |
| Other | 19 (13.6%) | 121 | |
| Race | | | |
| White | 133 (13.9%) | 826 | —[b] |
| Black | 1 | 2 | |
| Other | 1 | 2 | |
| Parity | | | 0.375 |
| 0 | 55 (15.3%) | 305 | |
| 1–2 | 69 (12.9%) | 467 | |
| 3–4 | 12 (16.9%) | 59 | |
| ≥5 | 0 | 6 | |
| Prior miscarriage | | | |
| Yes | 29 (9.8%) | 266 | 0.012 |
| No | 107 (15.8%) | 571 | |
| Prior PTL | | | |
| Yes | 9 (15.0%) | 51 | 0.847 |
| No | 127 (13.9%) | 786 | |
| Prior PTD | | | |
| Yes | 9 (14.3%) | 54 | 0.999 |
| No | 127 (14.0%) | 783 | |
| Prior PPROM | | | |
| Yes | 6 (11.5%) | 46 | 0.687 |
| No | 130 (14.1%) | 791 | |
| Tobacco use | | | |
| Yes | 15 (8.4%) | 164 | 0.013 |
| No | 121 (15.3%) | 669 | |
| ETOH use | | | |
| Yes | 7 (10.3%) | 61 | 0.379 |
| No | 128 (14.2%) | 771 | |

[a]PTL, preterm labor; PTD, preterm delivery; PRROM, preterm premature rupture of the membranes.
[b]Not tested due to small numbers.
*Probability from chi-square test of equal proportions.

found to be positive for GBS on at least 1 culture, only 28 (3.8%) were positive on all 4 cultures. In 143 patients (19.5%) GBS carriage was transient or intermittent. Transient carriers were defined as having only 1 of 4 cultures positive. Fifty-seven subjects (7.8%) were positive in 1 culture only, with 11 (1.5%) of these being positive only on the delivery culture. Intermittent carriers were defined as those with 1–3 of the 4 cultures positive. Of the intermittent carriers, 46 (6.2%) had 2 of 4 cultures positive for GBS, and 40 (5.5%) were positive for GBS in 3 of 4 cultures. Table 5 illustrates the varied patterns of GBS carriage which occurred.

Positive GBS culture results were separated by grade (light, moderate, and heavy carriage), as illustrated in Table 6 for the 26–28 week and labor cultures. Logistic regression analysis revealed that the prediction of positive delivery cultures was improved little, if at all, by using the full graded scale. This result follows from the fact that all positive grades showed similar colonization rates at delivery (Table 7).

### DISCUSSION

Great efforts have been expended by the obstetric and pediatric communities as well as the Centers for Disease Control (CDC) to develop strategies for the prevention of perinatal GBS morbidity and mortality.[11,16–18] What role, if any, antenatal GBS cultures should play in such strategies has been controversial due to the lack of clinical trials justifying their benefit. The Marshfield Clinic provides obstetric services for a rural population of central and northern Wisconsin. Our study was designed to provide data on the utility of antenatal GBS cultures in a population such as ours by determining the GBS prevalence, carriage patterns, and predictability of culture status at delivery.

Sample size calculations for planning this study assumed a prevalence of 15%, and would provide adequate statistical power to distinguish culture sensitivities of 80% and 90% (a 10% difference). The observed prevalence was somewhat lower than that used for planning, and the observed sensitivities were much lower, reducing the power actually achieved. However, the actual sample size provided good estimates of prevalence (Table 1) and we were able to demonstrate a significant dif-

TABLE 5. GBS carriage patterns in patients with all 4 study cultures (N = 735)

| Code[a] | Frequency | % |
|---|---|---|
| ++++ | 28 | 3.8 |
| +++− | 13 | 1.8 |
| ++−+ | 7 | 1.0 |
| ++−− | 9 | 1.2 |
| +−++ | 4 | 0.5 |
| +−+− | 6 | 0.8 |
| +−−+ | 3 | 0.4 |
| +−−− | 27 | 3.7 |
| −+++ | 16 | 2.2 |
| −++− | 8 | 1.1 |
| −+−+ | 11 | 1.5 |
| −+−− | 11 | 1.5 |
| −−++ | 9 | 1.2 |
| −−+− | 8 | 1.1 |
| −−−+ | 11 | 1.5 |
| −−−− | 564 | 76.7 |

[a]Code: first trimester/26–28 weeks/37 weeks/delivery.

TABLE 6. Graded culture results at 26–28 weeks vs. labor

| | Labor culture result | | | | |
|---|---|---|---|---|---|
| | Negative | Light | Moderate | Heavy | Total |
| Graded 26–28 week result | | | | | |
| Negative | 675 | 20 | 7 | 4 | 706 |
| Light | 21 | 15 | 12 | 4 | 52 |
| Moderate | 14 | 9 | 10 | 4 | 37 |
| Heavy | 9 | 6 | 5 | 2 | 22 |
| Total | 719 | 50 | 34 | 14 | 817 |

TABLE 7. Graded results at 26–28 weeks vs. percent positive at labor

| | Labor culture result | |
|---|---|---|
| | N | % Positive |
| Graded 26–28 week result | | |
| Negative | 706 | 4.4 |
| Light | 52 | 59.6 |
| Moderate | 37 | 62.2 |
| Heavy | 22 | 59.1 |
| Overall | 817 | 12.0 |

ference in the sensitivity of cultures in the first trimester compared with cultures obtained later. The observed sensitivities at all culture times were, however, so low (<70%, Table 3) as to limit their usefulness as screening tools. Despite the low observed sensitivities, our study did, however, confirm the value of a negative culture at 37 weeks (NPV of 95%), with approximately 5% of women missing treatment in labor. This NPV is similar to those described by Boyer et al.[3,19] and Yancey et al.[6] when the 95% confidence limits are considered.

Not surprisingly, first trimester cultures in our patients were not useful predictors of culture status at delivery. However, based on other published data, we anticipated that the closer to term cultures were obtained the better predictors they would serve with respect to culture status at delivery.[3,6,19] Boyer et al.[3] found a GBS culture utilizing broth enrichment at 28 weeks to have a sensitivity of 70% with respect to culture status at delivery and almost 100% positive predictability if done within 5 weeks of delivery in an inner-city population where the GBS prevalence was 23%. In a large multicenter study, however, utilizing a similar culture technique, it was found that cultures obtained at 23–26 weeks were not reliable predictors of GBS carriage at delivery.[20] Our data revealed comparable numbers with respect to the 26–28 week culture, but obtaining the culture at 37 weeks did not improve predictive values. The PPV of cultures at both 26–28 and 37 weeks was, however, poor. Nearly 40% of women culture positive at delivery and thus candidates for chemoprophylaxis would be missed if only those positive at 26–28 weeks or at 37 weeks were treated during labor. Uniformity in the method of obtaining cultures from our patients was closely monitored during the course of the study and therefore we do not believe a breech in technique accounts for this. Rather, we suspect the relatively low GBS prevalence in our population to at least in part be the explanation. The lack of diversity in our population may affect the prevalence, but it should not affect the properties (sensitivity and specificity) of the tests. No relatively common patient characteristics are apparent in our population base which could make the test less sensitive, or cultures more difficult to collect. In fact, we speculate that the lack of diversity may actually allow for a more accurate appraisal of the results due to the lack of other unsuspected or unrecognized confounding variables in a more heterogeneous population. Studies have shown enhanced detection of GBS with the use of selective broth media, therefore our laboratories' use of selective solid media for culturing could have impacted on our ability to isolate GBS and thus result in a falsely low prevalence in our population.[21] Recently,

Yancey et al.[6] examined the PPV of late anogenital GBS cultures utilizing broth enrichment and found a PPV of 87% within 5 weeks of delivery.

The natural history of GBS carriage in a rural obstetric population has been ascertained from this study. Although GBS carriage in pregnancy did not vary significantly by trimester, how individual patients carried GBS was a highly variable event. The identification that GBS carriage may be transient, intermittent, or consistent supports other published reports.[5]

Maternal-infant GBS transmission can occur even with light vaginal GBS colonization. However, the density of maternal GBS colonization has been shown to be an important predictor of transmission to the infant, with the highest rates of transmission in those who are heavily colonized.[10] We attempted to determine if grading of GBS cultures would be helpful in predicting culture status at delivery and consistent carriage in pregnancy. In our population, grading culture results (i.e., light, moderate, heavy) did not improve the prediction of culture status at delivery. However, over half of positive cultures showed light colonization and with roughly 100 GBS positive women to evaluate our ability to investigate the value of grading was limited. Thus, grading GBS cultures was not helpful in our data set, but we cannot conclude that grading has no value.

The cost effectiveness of universal screening remains a major concern particularly in light of the varied GBS prevalence in different patient populations. An effective rapid screen for GBS would obviate these problems but no such test is presently available. The recent CDC guidelines have recommended that a strategy for GBS disease prevention be employed whereby intrapartum antibiotic therapy is based on risk factors for disease or based on a positive GBS culture obtained at 35–37 weeks.[17] The American College of Obstetrics and Gynecology (ACOG) supported the recent CDC publication but cautioned that limited data on the utility of GBS antenatal cultures have been published.[18] We believe that we have shown that in a population with a relatively low GBS carriage rate, antenatal cultures utilizing selective solid media are poor predictors of GBS carriage at delivery regardless of when they are done. As part of our study an intrapartum treatment protocol based on antenatal cultures obtained at 37 weeks was also conducted. Results of that treatment study and cost analysis will be the subject of a separate paper. At present, however, because of our study findings, our current approach is to base intrapartum chemoprophylaxis on clinical risk factors rather than antepartum cultures.

### REFERENCES

1. Zangwill KM, Schuchat A, Wenger JD: Group B streptococcal disease in the United States, 1990: Report from a multistate active surveillance system. MMWR 41:25–32, 1990.
2. Weisman LE, Stoll BJ, Cruess DF, Hall RT, Merenstein GB, Hemming VG: Early onset group B streptococcal sepsis: A current assessment. J Pediatr 121:428–433, 1992.
3. Boyer KM, Gadzala CA, Kelly PD, Burd LI, Gotoff SP: Selective intrapartum chemoprophylaxis of neonatal group B streptococcal early-onset disease. II. Predictive value of prenatal cultures. J Infect Dis 148:802–809, 1983.
4. Dillon HC, Gray E, Pass MA, Gray BM: Anorectal and vaginal carriage of group B streptococci during pregnancy. J Infect Dis 145:794–799, 1982.
5. Anthony BF, Eisenstadt R, Carter J, Kim KS, Hobel CJ: Genital and intestinal carriage of group B streptococci during pregnancy. J Infect Dis 143:761–766, 1981.
6. Yancey MK, Schuchat A, Brown LK, Ventura VL, Markenson GR: The accuracy of late antenatal screening cultures in predicting genital group B streptococcal colonization at delivery. Obstet Gynecol 88:811, 1996.
7. Baker CJ, Edwards MS: Group B streptococcal infections. In: Remington TA, Klein JO (eds): Infectious Diseases of the Fetus and Newborn Infant. 4th ed. Philadelphia: W.B. Saunders, pp 980–1054, 1995.
8. Boyer KM, Gadzala CA, Burd LI, Fisher DE, Paton JB, Gotoff SP: Selective intrapartum chemoprophylaxis of neonatal group B streptococcal early-onset disease. I. Epidemiologic rationale. J Infect Dis 148:795–801, 1983.
9. Yancey MK, Duff P, Clark P, Kurtzer T, Frentzen BH, Kubilis P: Peripartum infection associated with vaginal group B streptococcal colonization. Obstet Gynecol 84: 816–819, 1994.
10. Boyer KM, Gadzala CA, Kelly PD, Gotoff SP: Selective intrapartum chemoprophylaxis of neonatal group B streptococcal early-onset disease. III. Interruption of mother-to-infant transmission. J Infect Dis 148:810–816, 1983.
11. American College of Obstetrics and Gynecology: Group B streptococcal infections in pregnancy. ACOG Tech Bull 170:1–5, 1992.
12. Towers CV, Garite TJ, Friedman WW, Pircon RA, Nageotte MP: Comparison of a rapid enzyme-linked immunosorbent assay test and the Gram stain for detection of group B streptococcus in high-risk antepartum patients. Am J Obstet Gynecol 163:965–967, 1990.

13. Armer T, Clark P, Duff P, Saravanos K: Rapid intrapartum detection of group B streptococcal colonization with an enzyme immunoassay. Am J Obstet Gynecol 168:39–43, 1993.
14. Anthony BF, Okada DM, Hobel CJ: Epidemiology of group B streptococcus: Longitudinal observations during pregnancy. J Infect Dis 137:524–530, 1978.
15. Hoogkamp-Korstanje JAA, Gerards LJ, Cats BP: Maternal carriage and neonatal acquisition of group B streptococci. J Infect Dis 145:800–803, 1982.
16. Committee on Infectious Diseases and Committee on Fetus and Newborn: Guidelines for prevention of group B streptococcal (GBS) infection by chemoprophylaxis. Pediatrics 90:776–777, 1992.
17. Centers for Disease Control and Prevention: Prevention of perinatal group B streptococcal disease: A public health perspective. MMWR 45(RR-7):1–24, 1996.
18. American College of Obstetrics and Gynecology Committee Opinion: Prevention of early-onset group B streptococcal disease in newborns. 173:1–8, 1996.
19. Boyer KM, Gotoff SP: Prevention of early-onset neonatal group B streptococcal disease with selective intrapartum chemoprophylaxis. N Engl J Med 314:1665–1669, 1986.
20. Regan JA, Klebanoff MA, Nugent RP, Eschenbach DA, Blackwelder WC, Lou Y, et al.: Colonization with group B streptococci in pregnancy and adverse outcome. Am J Obstet Gynecol 174:1354–1360, 1996.
21. Philipson EH, Palermino DA, Robinson A: Enhanced antenatal detection of group B streptococcus colonization. Obstet Gynecol 437, 1995.
